# Tetrodotoxin as a Tool to Elucidate Sensory Transduction Mechanisms: The Case for the Arterial Chemoreceptors of the Carotid Body

**DOI:** 10.3390/md9122683

**Published:** 2011-12-15

**Authors:** Asuncion Rocher, Ana Isabel Caceres, Ana Obeso, Constancio Gonzalez

**Affiliations:** Department of Biochemistry and Molecular Biology and Physiology, School of Medicine-IBGM, CIBER of Respiratory Diseases, ISCiii, University of Valladolid, 47005-Valladolid, Spain; Email: ana.caceres@yale.edu (A.I.C.); aobeso@ibgm.uva.es (A.O.); constanc@ibgm.uva.es (C.G.)

**Keywords:** carotid body, O_2_-sensing, tetrodotoxin, TTX, veratridine, dihydropyridine, catecholamine

## Abstract

Carotid bodies (CBs) are secondary sensory receptors in which the sensing elements, chemoreceptor cells, are activated by decreases in arterial PO_2_ (hypoxic hypoxia). Upon activation, chemoreceptor cells (also known as Type I and glomus cells) increase their rate of release of neurotransmitters that drive the sensory activity in the carotid sinus nerve (CSN) which ends in the brain stem where reflex responses are coordinated. When challenged with hypoxic hypoxia, the physiopathologically most relevant stimulus to the CBs, they are activated and initiate ventilatory and cardiocirculatory reflexes. Reflex increase in minute volume ventilation promotes CO_2_ removal from alveoli and a decrease in alveolar PCO_2_ ensues. Reduced alveolar PCO_2_ makes possible alveolar and arterial PO_2_ to increase minimizing the intensity of hypoxia. The ventilatory effect, in conjunction the cardiocirculatory components of the CB chemoreflex, tend to maintain an adequate supply of oxygen to the tissues. The CB has been the focus of attention since the discovery of its nature as a sensory organ by de Castro (1928) and the discovery of its function as the origin of ventilatory reflexes by Heymans group (1930). A great deal of effort has been focused on the study of the mechanisms involved in O_2_ detection. This review is devoted to this topic, mechanisms of oxygen sensing. Starting from a summary of the main theories evolving through the years, we will emphasize the nature and significance of the findings obtained with veratridine and tetrodotoxin (TTX) in the genesis of current models of O_2_-sensing.

## 1. Introduction: A Summary of Carotid Body (CB) General Function and Mechanisms up to the Mid-1980s

The carotid bodies (CBs) were discovered towards the middle of eighteen century, and thought for many years to be a small ganglion of the vegetative or autonomic nervous system. Accordingly, the CB received the names of ganglium parvum, ganglium minutum, ganglium exiguum or gangliolum intercaroticum. Early in the second half of the nineteen century, the great German anatomist Hubert von Luschka redefined the apparent nature of the CB, and proposed that the CB was not a ganglium but an endocrine gland, that he named glandula intercarotica (readers interested in early historical aspects are referred to [1]). The great prestige of the German anatomist meant that the CB was considered to be an endocrine gland, glandula intercarotica or carotid gland to almost the middle of the twentieth century. In a brilliant study on the innervation of the CB, Fernando de Castro, a favorite disciple of Cajal, discovered that the CB was neither a ganglion nor a gland, but a sensory organ as it received sensory innervation via the carotid sinus nerve (CSN) from sensory neurons located in the sensory ganglion (the petrosal ganglion) of the IX cranial nerve (the glossopharyngeal). A few years later, Heymans and coworkers discovered the function of the CB as the origin of cardiorespiratory reflexes and was awarded the Nobel Prize in 1938 [2].We cite this exact quote in the note as our tribute of recognition to Fernando de Castro’s role in the discovery of arterial chemoreceptors; regrettably his contribution was not awarded. 

The CBs are small paired organs located in the vicinity of the carotid artery bifurcation, existing as minor anatomical variations between species and among individuals of the same species. It is commonly pear or cone-shaped with the apex cephalically oriented and the base resting on the carotid sinus or one of the large arteries of the region. It is surrounded by a thin capsule of loose connective tissue which contains a dense net of small veins, giving the organ a distinct reddish-pink appearance that facilitates its recognition. Occasionally with the naked eye, although quite frequently a dissecting microscope is needed, the entrance into the organ can be seen by its cephalic pole of a thin nerve, the carotid sinus nerve (CSN a branch of the glossopharyngeal or IX cranial nerve) which represents the sensory innervation of the CB. Somas of the CSN fibers are located in the petrosal ganglion, the sensory ganglion of the glossopharyngeal. A section of a well perfused CB allows us to distinguish its basic structure [3]: thin walls of connective tissue containing a dense net of capillaries and venules emerge from the external capsule and divide the CB interior in lobules. Each lobule houses a cluster of cells of two types: rounded or ovoid chemoreceptor cells (also known as type I or glomus cells) are located towards the center of the cluster and flatted disc-shaped substentacular cells (or type II) are located towards the periphery of clusters. With appropriate stains it is possible to see bundles of nerve fibers travelling in the connective tissue walls and penetrating the cell clusters to form polymorphic endings in close apposition to chemoreceptor cells, forming the “sensory synapse” of the CB. In ultrathin sections it is possible to see a dense population of dense-core vesicles, which represent the most conspicuous trait of chemoreceptor cells. These vesicles contain catecholamine (and ATP and some neuropeptides) are commonly seen clustered facing sensory nerve endings. Chemoreceptor cells also contain clear-core vesicles that are thought to contain acetylcholine. 

The CB is a secondary sensory receptor in which the sensing elements, chemoreceptor cells, are activated by decreases in PO_2_ (hypoxic hypoxia) and increases in PCO_2_ and [H^+^] in arterial blood. The location of CBs in vicinity of the bifurcation of the common carotid artery has commonly been seen as if the CBs were tasting blood reaching the brain for possible correction of its composition. Upon activation, chemoreceptor cells increase their rate of release of neurotransmitters which drive the sensory activity in the CSN, which ends in the nucleus of tractus solitarius in the brainstem where the first synaptic station of the CB chemoreflex is located. Fibers from neurons located in the nucleus of the tractus solitarius project to several nuclei of the medulla and pons to ultimately generate the efferent orders of the chemoreflex. These efferent orders are directed to the motoneurons of the respiratory muscles and to sympathetic outflow neurons in the spinal cord to control the intensity/frequency of the respiratory movements and several cardiovascular parameters (heart frequency, vascular resistance, *etc*.) and adrenal medulla secretion of catecholamines (CA). Additional neural information entering brainstem from the CB ascends from the nucleus tractus solitarius to the hypothalamus to control the secretion of several hormones (ACTH-cortisol, vasopressin) whose plasma levels change during hypoxia and hypercapnia/acidosis; these hormones also contribute to the overall adaptive and homeostatic function of the CB [3,4,5]. The overall reflex responses originated in the CB are: an increase in lung ventilation in response to hypoxia and hypercapnia, bradycardia with increased cardiac output due to a positive inotropic effect and an increase in systemic blood pressure due to increases in resistance in most vascular beds, increase in the release of CA, adrenocorticotropic hormone-cortisol, antidiuretic hormone, and renin, and thereby angiotensin II.

**Figure 1:**
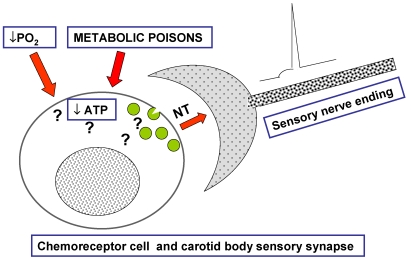
By the middle 1970s, the carotid body (CB) remained as a mystery. The theory most commonly accepted had been proposed by Anichkov and Belenkii in 1963 [17]. It was based on the observation that metabolic poisons, which were known to decrease ATP levels in cells, excited the CB increasing carotid sinus nerve (CSN) activity and eliciting brisk ventilatory responses. The reasoning was that hypoxia would act alike metabolic poisons, but no test was provided on whether mild hypoxia that excites the CB do in fact decrease ATP and no mechanisms were proposed as to how the ATP decrease would be coupled to the exocytosis of neurotransmitters [3].

Even if the significance of the CB at the systemic level was reasonably well characterized by the middle of the second half of the past century, the cellular mechanisms operating in the CB, *i.e.*, how chemoreceptor cells detect the stimuli (sensory transduction) and the synaptic mechanisms communicating chemoreceptor cells and the sensory nerve endings of the CSN, that is, the sensory neurotransmission mediating conducted electrogenesis in his secondary sensory receptor [6,7] remained unknown. In 1977, Gonzalez and Fidone [8] directly measured, for the first time, the release of catecholamines (CA), mostly dopamine (DA), by chemoreceptor cells of the rabbit CB when challenged with hypoxia. In successive studies [9,10], we firmly established that release of CA from chemoreceptor cells occurred in a close relationship with intensity of hypoxic and hypercapnic stimuli and in proportion to the action potential frequency in the CSN. Taking into account that petrosal ganglion neurons express catecholaminergic traits [11] and when cultured they release CA in response to several stimuli [12], it might be argued that our supposedly chemoreceptor cell CA release is “contaminated” by the release from intraglomic sensory nerve endings. Although such contribution exists, quantitatively speaking is so small that it is negligible: peak CA release obtained in response to supramaximal electrical stimulation of the carotid sinus nerve was 2.5 pmol/g CB tissue/5 min [13] and peak release in response to a hypoxic stimulus of intermediate intensity was 400 pmol/g CB tissue/5 min [9]. Additionally, a potential short-term acute modulation of chemoreceptor cells function by other neurotransmitters contained in the sensory nerve endings [4] was demonstrated [9], but the relationship between hypoxic stimulation and release was maintained in deafferented CBs. Overall, our observations on the release of CA from chemoreceptor cells drove our laboratory to focus the problem of sensory transduction in these cells, not as a unique or unparalleled process (Figure 1), but rather as a common process occurring in a great variety of secretory cells and presynaptic nerve endings. In other words, we started to experimentally approach sensory transduction in the CB as a stimulus-secretion coupling process in the same manner that Douglas and Rubin [14,15] and Katz [16] did while working, respectively, in the secretion of CA in the adrenal medulla and in the neuromuscular junction. 

Despite the notions of the uniqueness of the CB, the difficulty in studying this O_2_-CO_2_ sensing organ, and the also small chemoreceptor cells meant it pervaded the literature. For example, even if the early 1980s saw published works [8,9,10,18] demonstrating the calcium dependency of the release of CA elicited by natural stimuli, therefore conforming the stimulus-secretion coupling process, it was still undecided whether or not the membrane potential of chemoreceptor cells was dependent on K^+^ ion. In a seminal paper from our laboratory in 1986 by Almaraz *et al.* [19] it was concluded: “…our study shows that high extracellular K^+^ evokes a Ca^2+^-dependent secretory response in chemoreceptor cells with a threshold and dose-response expression not different from that obtained in a variety of structures where high K^+^ has been shown to depolarize their membrane. This suggests that the membrane potential of type I cells is dependent on the extracellular concentration of this ion. The secretory response also follows the general Ca^2+^ theory of neurosecretion. Finally…the fact that natural stimuli for the CB chemoreceptors elicit a release of DA which also is Ca^2+^ dependent makes it of interest to further characterize Ca^2+^ conductances involved in the secretory response”. 

Another finding indicating that hypoxia depolarized chemoreceptor cells was the increase of glucose consumption in the CB induced by hypoxia (the natural stimulus to chemoreceptor cells) of low intensity. This increase was tissue specific and ouabain sensitive [20,21,22], implying was that hypoxia should depolarize chemoreceptor cells and that depolarization would, at least in part, due to Na^+^ entry into the cells [23]; Na^+^ entering would activate the Na^+^/K^+^-dependent ATPase and thereby explain the sensitivity of the increased glucose consumption to ouabain. In other words, if the data with K^+^ suggested that hypoxia should depolarize to activate the voltage dependent Ca^2+^ channels, the metabolic data suggested the involvement of Na^+^ channels in the depolarization process. Before presenting how we dealt with investigation of depolarization and definition of pathways for Ca^2+^ entry in this precise historical manner we will make a parenthesis to present the tools used: veratridine and tetrodotoxin and dihydropyridine.

## 2. The Tools: Veratridine and Tetrodotoxin (TTX), and Ca^2+^ Antagonists (Dihydropyridines)

We knew from the studies carried out in many endocrine glands, in particular the adrenal medulla [24] and in motor nerve endings that well respected scientists have been routinely using veratridine and TTX. The more recently introduced calcium channel antagonists, in particular of the dihydropyridine family, entered into play in the neurotransmission-hormone secretion fields a few years later [25].

In his vast review of 1974 [26], Narahashi described the following traits for veratridine (an alkaloid derived from the family of Liliaceae): (1) its use in the study of excitable membranes has been limited, partly because its mechanism of action still remains to be fully elucidated. (2) It depolarizes through a change in a transient conductance and a selective increase in resting sodium permeability. (3) Its depolarizing action is eliminated in the absence of sodium and is antagonized by TTX. (4) Like high external K^+^ it promotes accumulation of calcium and release of neurotransmitters in synaptosomes, but while the effects of veratridine are blocked by TTX those of high K^+^ are not. (5) The association between calcium entry and neurotransmitter release induced by veratridine conforms the stimulus-secretion mechanism. In the 1984 edition of Hille’s book [27] there were no additional hints as to the mechanism of action of veratridine, but neurochemists and pharmacologists were enlarging the body of information regarding the effects of veratridine and its usefulness to study the stimulus-coupling process. For example, the studies by Kirpekar and co-workers [28] extended to isolated chromaffin cells and hypogastric nerve the initial observations on the release process induced by veratridine. Kirsnher laboratory working with isolated chromaffin cells used profusely veratridine in the experiments that allowed him to create the concepts of vesicular-quantal release of CA and of co-storage of classical neurotransmitters and neuropetides [29,30]. Kirshner laboratory generated a body of doctrine regarding veratridine action on neurosecretion that continues to be valid nowadays. On the biophysical side, the use of electrophysiological recordings, the measurements of ion fluxes, and the binding techniques [31,32] drove the knowledge regarding molecular mechanism of action of veratridine to levels overridden only when the explosion of molecular biology techniques appeared [33]. In any case, by the very early eighties it was firmly established that if in any given cell veratridine promotes a physiological response that is sodium-dependent and blocked by TTX, such a given cell possesses voltage-dependent Na^+^ channels.

TTX name derives from Tetraodontiformes, the name of the order that includes among other the pufferfish which carries the toxin in its liver and other internal organs. In the above-mentioned review by Narahashi, it is attributed the discovery and naming of TTX in the modern era to Tahara, in 1910 [34], although the same author had already presented some toxicological studies to the Pharmaceutical Society of Japan in 1889. Half a century later, Narahashi *et al.* in 1960 [35] in an elegant study carried out in frog skeletal muscle fibers and using microelectrodes that concluded: from the observations of resting potential, action potential, membrane resistance, and delayed rectification, TTX blocks action potentials by a selective inhibition of the transient (sodium) conductance normally occurring upon depolarizing stimulation. This study and its conclusion was the pillar of our understanding on the mechanism of action of TTX. Studies by Narahashi and many other researchers in the most classical preparations (squid axons, frog skeletal muscle, neuromuscular junction of the frog, squid axon synapse, Ranvier node, *etc*.) had produced according to PubMed 1089 publications by the time the review by Narahashi appeared in 1974. In those early days in the use of TTX, so many basic aspects of physiology were discovered with the judicious use of TTX, that we cannot resist quoting some: (1) Loewenstein *et al.* [36] concluded: “evidence is presented that spike and transducer processes in sensory receptors are independent events; impulse activity in tile crustacean stretch receptor neuron and the mammalian paccinian corpuscle was selectively blocked by a compound (tetrodotoxin) without affecting any of the parameters of the generator potential”. (2) Hagiwara and Nakayima [37] concluded: “Tetrodotoxin, … has no effect on the calcium action potential … of frog cardiac ventricle, tetrodotoxin suppresses the rate of rise of the action potential without affecting the overshoot; the suppressive effect of manganese ion is mainly on the overshoot of the action potential. This suggests that, in the action potential of the cardiac ventricle of the frog, the plateau phase is related primarily to the increase in permeability of the membrane to calcium ions”. (3) Katsuki *et al.* [38] working on guinea pig cochlea concluded: “Tetraethylammonium chloride … believed to decrease potassium conductance, and tetrodotoxin, which apparently decreases sodium conductance …. The former depressed the direct-current endocochlear potential and also the alternating-current cochlear microphonics (the receptor potential of the ear), but tetrodotoxin was ineffective except on the nerve impulses”. (4) Nonomura *et al.* [39] concluded: “Tetrodotoxin … has no effect on the spontaneous discharge in the smooth muscle of taenia coli … *[that was]* abolished by Mn^++^. Because Mn^++^ is a specific suppressor of the spike induced by Ca^++^ and tetrodotoxin is an inhibitor of the spike induced by Na^+^, we suggest that Ca^++^ is a charge carrier in the production of spike potential in the smooth muscle and that the entry of intervening Ca^++^ through the membrane acts as a trigger for the contraction of smooth muscle”.

In summary, before the arrival of the 1980s, the doctrine was firmly established: TTX is a very selective blocker of voltage-dependent Na^+^ channels that does not affect other ionic conductances and that has proven to be an extraordinary tool to establish many basic principles of physiology, including many aspects of stimulus secretion coupling and postsynaptic actions of neurotransmitters. But attaching to our interest in present article, the conclusion could be that the pufferfish toxin activity or efficacy to suppress the effects resulting from the stimulation of any cell type, whether physiologically or pharmacologically stimulated as with veratridine (but not depolarized by K^+^), was an univocal demonstration of the presence of voltage dependent sodium channel in the cell under study. 

Calcium antagonists [40] or “slow channel inhibitors” [41] were developed around the heart pathology, as potential treatments for heart ischemia or stroke and hypertension. In his early historical review of 1983, Fleckenstein [40] wrote that verapamil and prenylamine (both introduced in 1964), mimicked the cardiac effects of simple Ca^++^ withdrawal (they diminished Ca^++^-dependent ATP utilization, contractile force, and O_2_ requirement of the beating heart without affecting the Na^+^-dependent parameters of the cardiac action potential), and that their effects could easily be distinguished from those of β-blockers with simple experimental tools. Other substances that met the criteria of calcium antagonists in those days included D-600 (introduced in 1968) and the dihydropyridines nifedipine, niludipine and nimodipine (made available along the 1970s). All of them, as well as diltiazem introduced by the Japanese, interfered with the uptake of labelled Ca^++^ into the myocardium and blocked excitation-contraction coupling of vascular smooth muscle. On the bases of voltage-clamp experiments the conclusion was reached that all these antagonists act as specific inhibitors of the slow trans sarcolemmal Ca^++^ influx.

However, findings with the use of calcium antagonists or slow channel inhibitors or organic blockers of calcium channels up to 1984, remained inexplicably confusing because in some preparations (e.g., synaptosomes; see next paragraph) several laboratories reported that these drugs would not prevent depolarization ^45^Ca^2+^ uptake or and neurotransmitter release [42,43]. As a consequence, the preferred tools to investigate the role of Ca^2+^ in any process were extracellular Ca^2+^ removal or the use of other divalent cations (most frequently Mg^2+^ or Mn^2+^), inorganic blockers of calcium channels. In 1983, a dihydropyridine agonist of calcium channel, BAY-K 8644, was introduced [44], and the combined use of BAY-K 8644 with the more classical dihydropyridine antagonists defined pairs of tools of enormous value to investigate the role of Ca^2+^ in the stimulus secretion coupling, both in endocrine and synaptic preparations. Quite soon two different laboratories using PC12 and native adrenomedullary chromaffin cells [25,45] used both agents alone or in combination and concluded that nitrendipine-nifedipine (and other Ca^2+^ antagonists) bind to Ca^2+^ channel and block the entry of Ca^2+^ into the cells abrogating Ca^2+^ dependent responses, Bay 8644 competes with the binding of the dihydropyridine antagonist, and when applied alone promotes Ca^2+^ entry and triggers Ca^2+^ dependent responses. The electrophysiological observations of Nilius [46] in cardiomyoctes sanctioned the neurochemical-pharmacological observations on secretory cells as he stated: “… novel dihydropyridine compound BAY-K 8644 … *augments* the maximum upstroke velocity V_max_ of slow action potentials in concentrations between 2 × 10^−9^ and 2 × 10^−7^ mol/L …. The increase in V_max_ could be antagonized by nifedipine in a similar range of concentrations …. At concentrations higher than 6 × 10^−7^ mol/L BAY-K produced oscillatory afterdepolarizations indicating a drug-induced Ca overload. All the experimental findings are consistent with the hypothesis that the novel dihydropyridine compound BAY-K 8644 activates Ca-channels in the mammalian ventricular myocardium”. 

**Figure 2:**
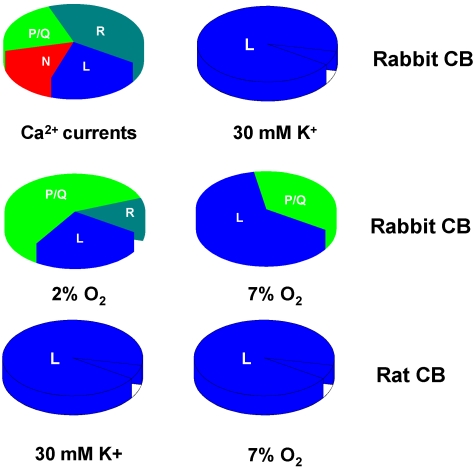
Relative contribution of different calcium channel subtypes to whole calcium current registered in primary cultures of chemoreceptor cells of the rabbit (Ca^2+^ currents) and in the secretory response elicited by high external K and hypoxia in *in vitro* CBs from rabbit and rat.

Therefore, a nice pair of tools had certainly been defined, but findings in synaptosomes stubbornly kept producing results that quite frequently did not fit the notions expressed in preceding paragraph (e.g., [47] and the conclusion in many of these articles was: results suggest that synaptosomal voltage-sensitive Ca^2+^ channels either are of a different type to those found in peripheral tissues including heart and smooth muscle cells and peripheral cells of neural origin …. Another typical conclusion was: “findings suggest that high-affinity binding of nitrendipine is not directly linked to voltage-dependent calcium uptake in brain” or “data indicate that high-affinity Ca^2+^ channel antagonist and agonist (nitrendipine and BAY-K 8644) binding sites exist in synaptosomal membranes but their relationship to functional Ca^2+^ channels is not clear. In the famous review by Hagiwara and Byerly of 1981 [48] unequivocal evidence was presented for the existence of different types of voltage dependent Ca^2+^ channels, even if the different subtypes of currents-channel have not been recorded in isolation. Tsien and coworkers in a series of articles between 1984–1986 summarized in McCleskey *et al.* [49] provided the direct experimental evidence for the diversity of Ca^2+^ channels, so that a single cell type might have several of those subtypes of channels, and thereby gave explanation to the conflicting results with the dihydropyridine agonists and antagonists of Ca^2+^ channels in synaptosomal, and occasionally in peripheral, preparations. Specific tools to dissect most of the channel subtypes role in physiological processes were soon after provided by the laboratory of Olivera in Salt Lake City [50] as they isolated many specific toxins for each channel subtype from marine snails and spiders. The dissection of Ca^2+^ channel subtypes in rabbit chemoreceptor cells has recently been done by our laboratory [51]. In brief, using current pharmacological-electrophysiological criteria for classification of Ca^2+^ we found that adult rabbit chemoreceptor cells exhibit components of their Ca^2+^ currents that are sensitive to ω-conotoxin GVIA and to ω-agatoxin IVA as well as to nisoldipine, suggesting the presence, in addition to L-type, of N and P/Q type Ca^2+^ channels as well as a significant residual component of R-channels. Their contribution to release of CA elicited by hypoxia and is shown in Figure 2. 

Interestingly, the involvement of the different subtypes of Ca^2+^ channels in the hypoxia-induced substance P release appears to be different. Thus, Kim *et al.* [52] found that about 85% of calcium dependent substance P release induced by hypoxia in the rabbit CB was inhibited by ω-conotoxin GVIA (an N-type Ca^2+^ channel blocker), while nitrendipine only inhibited the release by ~65%, and ω-agatoxin TK (a P/Q-type Ca^2+^-channel inhibitor) had no significant effect. 

In summary, by the middle of decade of the 1980 we have available a great armamentarium of specific tools to study the ionic properties of the membrane of chemoreceptor cells and certainly to explore the transduction of natural stimuli of chemoreceptor cells, hypoxia and hypercapnia, when it is conceptually seen as a stimulus-secretion process. 

## 3. The Advances in the Cellular Physiology of the CB 1984–1988 with the Use of Veratridine, TTX, and Dihydropyridines

Early in the introduction we referred to the names given to the CB in early times: all of them implying the small size of the organ (around 50, 350 and 500 µg in the rat, rabbit, and cat, respectively) and to the small size of chemoreceptor cells, ≤10 μm in diameter. To this it should be added that the CB is heterogeneous and contains several cell types with parenchymal chemoreceptor cells representing around 30% of the organ cells [4,53]. Obviously, this results in very poor yields of viable cells in early tentative dissociations and primary cultures of the CB; these facts, in turn, explain the running behind of the knowledge on CB cellular or general physiology.

These facts explain why our dealing with the problem of sensory transduction was based on the use of a classical neurochemical-pharmacological approach, tightly adhered to the stimulus secretion coupling doctrine. Those facts also explain why we adhere to measure DA, the reason being that in the CB, DA was specifically located in chemoreceptor cells, and why we used isotopic methods that provided the highest sensitivity. To these facts we needed to add an additional one: the use of very specific pharmacological tools as we have described in preceding paragraphs. Many of these basic principles were learnt by one of us while a postdoc in SJ Fidone’s laboratory in Salt Lake City but were fully developed in our laboratory in Valladolid, although the path between Valladolid and Salt Lake City has remained and still remains well in use in both directions [54,55,56]. 

Following experimental protocols used by Kirpekar and Garcia [57] we succeeded in demonstrating that nitrendipine greatly inhibited the release of DA induced by hypoxia in chemoreceptor cells and presented the findings in the VIIIth Meeting of the International Society for Arterial Chemoreception in the fall of 1985 [58,59,60]. During 1986, Rocher entered in our laboratory as a graduate student and quite soon she demonstrated that rabbit chemoreceptor cells were excitable in neurochemical-pharmacological terms because quite early she demonstrated that veratridine induced a Na^+^ and Ca^2+^ dependent release of DA from rabbit chemoreceptor cells. She showed further that veratridine induced release was nearly abolished by TTX, and most important, that low PO_2_-induced release also was partially inhibited by TTX. Her findings were communicated in early in 1987 to the Spanish Society of Biophysics, published as a short note in 1988 and as full length paper in 1994 [61,62,63]. Table 1 presents the status of our knowledge by the fall of 1987.

To the data of Table 1, which refer to rabbit CB chemoreceptor cells, we should add that TTX did not affect normoxic, basal ongoing release of DA. It should also be noted from Table 1 that even if Na^+^ channels participate in the cascade of events that occurs between the low PO_2_ detection and the neurosecretory response [63], this participation represents an amplifying signal because an important part of the response (≈60%) still remains when Na^+^ channels are fully inhibited. Additionally, Table 1 shows that the participation of dihydropyridine sensitive channels in the entry of Ca^2+^ to support hypoxia-induced release varies with the intensity of hypoxia, and finally, that even with mild hypoxia, some voltage-dependent pathways additional to L-type Ca^2+^ channels participate in the stimulus- secretion coupling, because at any given hypoxic intensity the evoked release of DA is Ca^2+^ dependent by more than 95%. Although chemoreceptor cells contain other neurotransmitters (ACh, ATP, opioid peptides, substance P, and probably others; [4]) that are known to be released by natural stimuli, we do not refer to them in the present context because neither veratridine or TTX have been used in the characterization of their release process.

**Table 1:** Neurochemical-pharmacological demonstration of the presence of voltage-dependent Na^+^ and L-type Calcium channels in chemoreceptor cells of freshly isolated rabbit carotid body.

Incubating Conditions	^3^H-CA Evoked Release (× Basal)	% Change
Veratridine (50 μM)	17.1 ± 0.9	---
Veratridine (50 μM) + TTX (1 μM)	0.3 ± 0.1	98 (inhibition)
Veratridine (50 μM), Na^+^-free	0	100 (inhibition)
Veratridine (50 μM), Ca^2+^-free	1.4 ± 0.3	92 (inhibition)
Hypoxia (10 % O_2_)	2.8 ± 0.95	---
Hypoxia (10 % O_2_) + TTX (1 μM)	1.7 ± 0.3	40 (inhibition)
Hypoxia (7% O_2_)	8.5 ± 2.2	---
Hypoxia (7% O_2_) + nisoldipine (0.625 μM)	1.9 ± 0.2	78 (inhibition)
Hypoxia (2% O_2_)	28.6 ± 3.3	---
Hypoxia (2% O_2_) + nisoldipine (0.625 μM)	17.1 ± 0.9	41 (inhibition)
Hypoxia (7% O_2_) + Bay K 8644 (1 μM)	31.2 ± 4.0	370 (potentiation)

Experimental results shown in Table 1 clarify a point, namely, that hypoxia is capable of activating voltage dependent ion Na^+^ and Ca^2+^ channels (and probably also K^+^ channels), and therefore, hypoxia must depolarize chemoreceptor cells. The emerging question has conceptual and methodological implications: how does hypoxia depolarize chemoreceptor cells? What are the ionic conductances involved? Electrophysiological methods were needed to go deep into the sensory transduction mechanisms in chemoreceptor cells. Yet, we want to state that, when advisable and feasible, we have kept combining electrophysiology and neurochemistry in our experimental designs in order to obtain the unitary or nearly hints on the mechanisms involved and the integrated view of the neurosecretory response [51].

## 4. Direct Electrophysiological Assessment of the Presence of Voltage Operated Channels in Rabbit Chemoreceptor Cells. The Discovery of O_2_-Sentive or O_2_-Regulated Channels and the Hypoxic Transduction Cascade

Classical intracellular recordings were extremely difficult due to the small size of the cells. As a consequence, the results they were yielding were difficult to interpret and conflicted with our own findings [3,64,65]. Therefore, it appeared that the electrophysiological approach to the problem of sensory transduction in the chemoreceptor cells of the CB must rely on the recently developed patch-clamp technique [66]. However, we had neither the technique, nor the very expensive equipment, nor the adequate preparation. Since we did not have the patch-clamp equipment or the expertise, after developing a primary culture of dissociated CB whose chemoreceptor cells retained functional properties in terms of metabolism of CA, we agreed on a collaboration with Lopez-Barneo in Sevilla, a well-established electrophysiologist. Several people from our laboratory spent almost the entire year of 1987 going to the slaughter house to get calf CBs that would produce a yield of chemoreceptor cells that we were able to isolate at near purity, in which we can measure ionic concentrations by flame photometry and the release of CA using radioisotopic method [67]. We switched to rabbit chemoreceptor cells and we obtained yields that we estimated adequate for the electrophysiological experiments. We succeeded and early in 1988, Lopez-Lopez travelled to Sevilla with required surgical instruments and enzyme batches and the recordings showed from the very first day that chemoreceptor cells were indeed excitable cells as they expressed Na^+^, Ca^2+^, and K^+^ voltage dependent currents. However, the resting Na^+^ conductance that we were hypothesizing that hypoxia would trigger/activate in accordance with the transduction in mechanoreceptors and the very recently described conductance gated by cAMP in olfactory receptors [68] was not there. The appearance in January 1988 of the paper by Avenet *et al.* [69] showing that sweet stimulus mediated increase in cAMP in taste receptor causes receptor depolarization via inhibition of K^+^ channels drove the attention to K^+^ currents and there it was: freshly isolated chemoreceptor cells of the rabbit CB have a component of their K^+^ currents that was reversibly inhibited by hypoxia [70]. Detailed characterization of the ionic currents in rabbit chemoreceptor cells appeared soon after in two full length studies [71,72]. In the decade that followed the initial description, several laboratories demonstrated the excitability of chemoreceptor cells in other species, e.g., [73,74]. All of them showed that, in addition to Ca^2+^ currents, chemoreceptor cells possess some component of their K^+^ conductances that was reversibly inhibited by hypoxia [73,74]. The presence of Na^+^ channels in rat chemoreceptor cells was a debated issue existing laboratories whose cells completely lack Na^+^ currents, while in others different percentages of cells expressed them [75]. Yet, it should be stated that Doyle and Donnelly [76] had reported that TTX inhibits by around 25% the release of CA elicited by anoxic stimuli. So the hypoxic sensory transduction process appeared as a simple cascade: hypoxia inhibits K^+^ currents and leads to chemoreceptor cell depolarization, activation of voltage dependent Ca^2+^ channels, and triggering the release of neurotransmitter that would drive sensory nerve endings of the CSN; activation of Na^+^ currents, if present, would amplify the signal to trigger Ca^2+^ channel opening. In more recent years, several laboratories have shown that mice chemoreceptor cells also possess some component of their Kv current that is reversibly inhibited by hypoxia [77,78], yet neither Na^+^ or Ca^2+^ currents have been described in the cells of this species. Surely we will undertake the more classical neurochemical-pharmacological approach to show if chemoreceptor cells naturally organized as they remain in the intact freshly isolated CBs have the channel armamentarium, defined with veratridine/TTX and dihydropyridine/conotoxins, as to generate inward currents. 

All the O_2_-sensitive currents so far described in chemoreceptor cells were voltage-dependent. This fact created a kind of muffled uneasiness: if the O_2_-sensitive currents are voltage-dependent it means that for them to become inhibited they must be activated by a prior depolarizing event. Gonzalez and co-workers [4,79] provided tentative and, might be imaginative answers, to this unsolved issue that, that we believe was experimentally resolved by Keith Buckler when, in 1997, he showed that rat chemoreceptor cells express, in addition to the O_2_-sensitive Ca^2+^ dependent K^+^ current described by Peers, a voltage independent leaky K^+^ current active at resting membrane potential and reversibly inhibited by hypoxia [80]. Today, it is our believe that it is almost universally accepted that this leaky current carried on by TASK-1, TASK-2, or TASK-3 homo or heteromerically assembled [81,82] is the trigger of the initial depolarization that would bring the membrane potential to the threshold for activation of voltage dependent channels. Yet, inhibition of O_2_ sensitive voltage-dependent K^+^ currents would remain an absolute requirement for cell activation because outward currents, unless they are inhibited, exceed in amplitude inward currents and depolarization of the cells would not proceed [83,84]. 

The next question is: what do ion channels have in chemoreceptor cells or what do chemoreceptor cells have to make some of their K^+^ channels O_2_-sensitve? Are channels themselves the O_2_-sensors? If yes, which are the channel domains or microdomains to give them the specificity of O_2_-sensors? And if no, what is the O_2_-sensor molecule present in chemoreceptor cells and functionally coupled to the O_2_-sensitive channels? Tentative, but not definitive, answers to these questions are beyond the present review and have been recently addressed by our and other laboratories [7,84,85,86].

## 5. The Significance of Na^+^ Channels in Rat Chemoreceptor Cell Responses to Hypoxia

As mentioned above, controversies regarding the expression of Na^+^ channels in rat chemoreceptor cells using exclusively electrophysiological approaches and the absence of data in freshly isolated CB preparations drove us to study the potential significance of these ion channels in rat chemoreceptor cells using neurochemical, pharmacological and molecular biology approaches [87]. Voltage-gated sodium channels (Na_v_) are large glycoproteins consisting of three subunits, one α subunit and two β subunits (β1 and β2) [33]. The α subunit (≈220 kDa) forms the voltage-gated sodium selective aqueous pore and the β subunits modify channel properties and interact with cytoskeletal and extracellular matrix proteins. To date, at least nine different α subunits genes have been identified in mammals, named SCN1-11 [88,89]. Pharmacologically, Na^+^ channel isoforms are commonly distinguished by their sensitivity to TTX: most sodium channels are blocked by nanomolar (IC_50_ ≤ 20 nM) concentrations of TTX and are defined as TTX-sensitive (TTX-s) while others, requiring micromolar TTX (IC_50_ ≥ 0.5 µM) concentrations for their blockade, are defined as TTX-resistant channels (TTX-r) [27]. 

**Figure 3:**
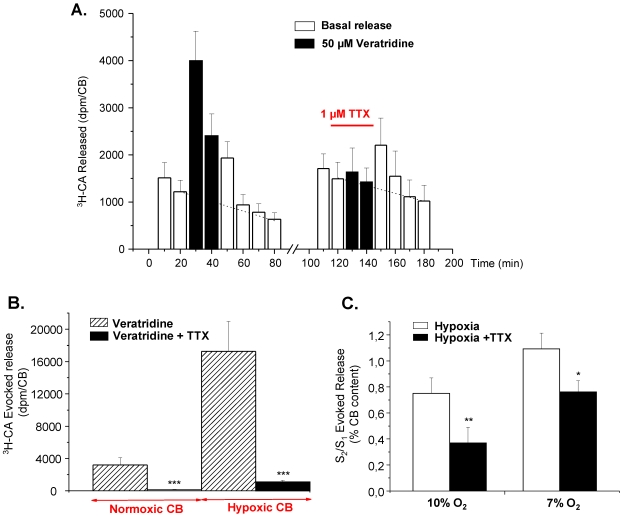
Effect of tetrodotoxin (TTX) on the release of ^3^H-catecholamines evoked by veratridine and hypoxia in rat CB. (**A**) Time course of veratridine effect (50 μM) on ^3^H-CA release and TTX inhibition; (**B**) Veratridine and TTX effect on ^3^H-CA evoked release from normoxic and chronically hypoxic CBs; (**C**) Effect of TTX on release of ^3^H-catecholamines elicited by graded hypoxia. Bars represent ratios of total evoked release obtained with second stimulus challenge to that obtained with first one (S_2_/S_1_) for control (open bars) and TTX treated CBs during the second stimulus challenge (filled bars). Values are means ± SEM for 6–10 CBs. Statistically significant differences: * *p* < 0.05; ** *p* < 0.01. (Adapted from [87] with permission).

We have used RT-PCR, immunoblotting, and immunocytochemical techniques to assess the presence of Na^+^ channels in the rat CB, to define their molecular identity and to locate them in chemoreceptor cells from adult rats. Additionally, we have used neurochemical and pharmacological techniques to define the functional significance of Na^+^ channels in the neurotransmitter release response to hypoxia from chemoreceptor cells in a preparation of freshly isolated intact rat CB. We have found that rat CB expresses five TTX-sensitive Na_v_ isoforms detected by RT-PCR in total RNA extracted from the intact organ. We didn’t explore the presence of TTX resistant channels because the release ^3^H-CA elicited by veratridine was inhibited by in full in the intact organ by 1 µM TTX, as they did omission of Na^+^ and Ca^2+^ from the incubating solutions. In immunocytochemical and Western blot experiments, carried out, respectively, in frozen sections of the entire CB and crude homogenate of the intact organ and using a polyclonal antibody capable of reacting with a sequence common to all Na_v_ α subunits, we provided unequivocal evidence for the presence of the conducting α subunit of Na^+^ channels in the TH-positive chemoreceptor cell clusters of the CB. Immunocytochemistry carried out in fresh cultured dissociated CB cells indicates that only the α subunits of Na_v_1.1, Na_v_1.3 and Na_v_1.6 (the most abundant isoforms at the mRNA level) are indeed expressed in all TH-positive cells (Figure 3). 

In the experiments presented in our study with the intact preparation of the rat CB, we clearly showed that Na^+^ channels in chemoreceptor contribute by nearly 50% to the DA release response elicited by hypoxic stimuli of low intensity, and by a smaller percentage to the release response elicited by more intense hypoxia (Figure 3C); [76]. Although even in the most favorable electrophysiological studies [90] the density of Na^+^ currents recorded from dissociated chemoreceptor cells is low in comparison to the density of Ca^2+^ currents, our data imply that Na^+^ currents contribute to increase the amplitude of the action potentials and therefore to the amount of Ca^2+^ entering the cells and to the magnitude of the Ca^2+^ dependent ^3^H-CA exocytosis. In this regard, Buckler and Vaughan-Jones [91] found that rat type I cells both under hypoxic stimulation can generate bursts of repetitive action potentials with clear overshoots suggestive of a contribution of Na^+^ currents in their genesis; higher overshoots are more evident in the action potentials generated by chemoreceptor cells cultured in hypoxia [92]. Interestingly, Buckler and Vaughan-Jones [93] observed a comparable spiking activity during hypercapnic stimulation of rat chemoreceptor cells, but TTX instead of producing the expected decrease in Ca^2+^ increase elicited by hypercapnia, caused a 50% increase in the Ca^2+^ response. This observation led them to conclude that, while the reason for this enhancement is not known, the result clearly shows that sodium-dependent action potentials are not required for the hypercapnic Ca^2+^ response [94].

In general, discrepancies in findings between our results and electrophysiological experiments could be explained by differences among studies using enzymatically dispersed cells and our preparation using mostly intact organs. Dissociation procedures involving enzymatic digestion for long periods of time can cause damage in α or β subunits of Na channels and be responsible for the different percentages of cells expressing Na^+^ currents, as it has been described in smooth muscle cells from mouse mesenteric artery [95].

## 6. Significance of Na^+^ Channels on Long Term Hypoxic Responses

**Figure 4:**
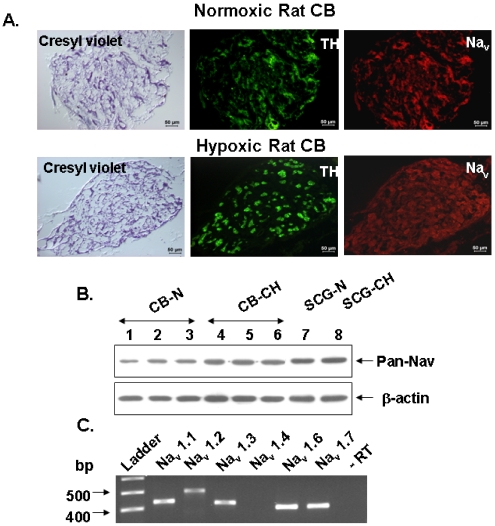
Expression of voltage-gated Na^+^ channel (Na_v_) in normoxic and chronically hypoxic rat CBs assessed by immunocitochemistry, Western Blot and RT-PCR. (**A**) Localization of Na^+^ channel isoforms Na_v_1.1, Na_v_1.3 and Na_v_1.6 by immunofluorescence in cultured rat chemoreceptor cells; (**B**) Representative immunoblot obtained from normoxic (CB-N; lanes 1–3) and chronic hypoxic (CB-CH; lanes 4–6) CBs homogenates fractionated on 8% SDS-polyacrilamide gel. Lanes 7 and 8 correspond to a positive control (superior cervical ganglion); (**C**) Agarose gel electrophoresis of different Na_v_ channel genes (upper panels) and β-actin (lower panel; housekeeping gene) and tyrosine hydroxylase (TH). (Adapted from [87], with permission).

Chronic hypoxia (CH) enhances chemosensitivity in the CB and induces extensive and specific changes in gene expression including modifications in the rate of expression and regulation of K^+^ and Ca^2+^ channels, which are associated with O_2_-sensing and neurotransmitter release. In this regard, pioneer studies from Hempleman [96] and Nurse’s group [90,92] suggested that *in vitro* applied CH may increase chemoreceptor cells excitability by increasing the depolarizing sodium current. In our study we observed that after 7-day exposure of rats to normobaric hypoxia (10% O_2_), the CBs became hypertrophic (50% increased weight), store enhanced amounts of CA, express increased rate of synthesis, and exhibit exaggerated rate release of CA when challenged with veratridine (×5 release in CBs of control rats) and by acute hypoxia (×3 release in CBs of control rats). As in control animals, in CH animals veratridine-evoked secretion was dependent on the presence of Ca^2+^ and Na^+^ in the extracellular medium and was entirely blocked by 1 μM tetrodotoxin (TTX). Secretory response evoked by mild hypoxia (7% O_2_ during 10 min) retained the TTX-sensitivity seen in control animals, implying that functional adaptation of CB to chronic hypoxia *in vivo*, requires augmentation of Na^+^ channel expression to allow the enhanced functional requirements seen in more integrated responses such as increased neurotransmitter release (Figure 4). This higher concentration of neurotransmitter would at the end generate the sensitization of the CB to hypoxia seen in early weeks after sustained hypoxic exposure [97,98] evidenced by a higher frequency of action potentials in the CSN and the higher ventilatory response to acute hypoxic tests [98]. 

## 7. Conclusions

1. The carotid bodies were defined as arterial chemoreceptors around the 1930s, and by the 1970s most of the reflexes originated the carotid bodies had been defined. However, at the cellular level, these chemoreceptor organs remained mysterious.

2. Since the structural organization of the carotid bodies is that of a secondary sensory receptor, it has been feasible to conceive the functioning of the organ within the frame of the stimulus-secretion concept: the natural stimuli to the receptors, hypoxia and hypercapnia, should elicit the release of neurotransmitters from chemoreceptor cells that would generate the conducted activity in the sensory nerve innervating the organs to drive the reflexes.

3. Based on this basic conception we started to measure the release of neurotransmitters elicited by natural stimuli and, using the adequate tools, we were able to define the basic mechanisms present in chemoreceptor cells and activated by different stimuli. 

4. Tetrodotoxin, veratridine and dihydropyridines proved to be basic and indispensable tools that allowed us to define chemoreceptor cells as excitable cells, and further to define that hypoxia depolarized the cells to generate the release of neurotransmitters conforming the most orthodox stimulus secretion coupling process.

5. The development of a primary culture of chemoreceptor cells and the application of patch-clamp techniques confirmed the observations, and allowed to discover the existence of K^+^ channels in chemoreceptor cells reversibly inhibited by hypoxia, implying that they were primary elements in the genesis of the chemoreceptor cells depolarization.

6. These results generated in the second half of the 1980 decade working with rabbit CBs have in more recent years been extended to all mammalian species studied. 
